# Acupuncture plus Tuina for chronic insomnia: A protocol of a systematic review and meta-analysis

**DOI:** 10.1097/MD.0000000000027927

**Published:** 2021-11-24

**Authors:** Weiwan Yang, Xiaole Guo, Qi Lu, Ting Pan, Haili Wang, Hongfeng Wang

**Affiliations:** a Department of Acupuncture and Tuina, Changchun University of Chinese Medicine, Changchun, China; b Department of Tuina, Changchun University of Chinese Medicine, Changchun, China.

**Keywords:** acupuncture, chronic insomnia, protocol, systematic review, Tuina

## Abstract

**Background:**

Insomnia is a common sleep disorder. It refers to a subjective feeling of dissatisfaction with sleep duration and quality that affects social functioning, even though there are appropriate opportunities and environments for sleep. The disease for a long time can easily cause physical and mental fatigue, anxiety, depression, and other symptoms. Anxiety, depression, and insomnia can worsen the condition. Acupuncture and Tuina therapy has been widely used in the treatment of chronic insomnia, and this study aimed to conduct a meta-analysis of acupuncture plus Tuina in the treatment of chronic insomnia to clarify its efficacy.

**Methods:**

The following databases will be searched: Web of Science, PubMed, Cochrane Library, Embase, and Medline databases. In addition, we will also collect 4 databases of China: China National Knowledge Infrastructure, China Biomedical Literature Database, VIP Database, and Wan-fang Database. We selected eligible studies published up to October 2021. We used Review Manager 5.4, provided by the Cochrane Collaborative Network for statistical analysis. Clinical randomized controlled trials related to acupuncture plus Tuina for chronic insomnia were included in this study. Language is limited to both Chinese and English languages. Study selection, data extraction, and study quality assessment were independently performed by 2 researchers. We then assessed the quality and risk of the included studies and observed the outcome indicators.

**Results:**

This study provides a high-quality synthesis to assess the effectiveness and safety of acupuncture plus Tuina for treating chronic insomnia.

**Conclusion:**

This systematic review will provide evidence to determine whether acupuncture plus Tuina is an effective and safe intervention for patients with chronic insomnia.

**Ethics and dissemination:**

The protocol of the systematic review does not require ethical approval because it does not involve humans. This article will be published in peer-reviewed journals and presented at relevant conferences.

**Registration number:**

INPLASY2021100115

## 1 Introduction

At present, the aging of society is increasingly prominent, and the widespread problem of insomnia has aroused the attention of researchers worldwide. Insomnia is a common sleep disorder.^[1]^ It refers to a subjective feeling of dissatisfaction with sleep duration and quality that affects social functioning, even though there are appropriate opportunities and environments for sleep.^[2]^ Although insomnia is not a serious disease, it often affects people's normal work, life, study, and health. Chronic insomnia brings long-term pain to patients, and even forms dependence on sedative sleeping drugs, and can induce or aggravate a variety of physical diseases. With the deepening of the understanding of insomnia in the medical field, insomnia has become the second largest epidemic mental disease in the world as a high-risk predictor of mental disorders.^[3]^ Moreover, it increases the risk of cardiovascular diseases, digestive tract diseases, and senile dementia, and even lead to malignant events.^[4–6]^

Although insomnia is common, its pathogenesis remains unclear. Modern neurobiological and psychological perspectives indicate that brain function changes and genetic, behavioral, cognitive, and emotional factors jointly participate in the occurrence and development of insomnia.^[7–9]^ For most people, insomnia will soon disappear as the trigger subsides and homeostasis is adjusted for recovery. If the factors causing insomnia cannot be eliminated, or improper treatment of insomnia can lead to short-term or short-term insomnia into chronic insomnia. During the progression of chronic insomnia, the body and cerebral cortex may gradually produce excessive arousal.^[10]^ Somatic over-arousal is mainly caused by hyperfunction of the thalamic–pituitary–adrenal axis and over-activation of the sympathetic nervous system. Patients present with increased heart rate, heart rate variability, and basal metabolic rate, which can lead to cortical over-arousal.^[11]^

As a complementary and alternative medicine, traditional Chinese medicine (TCM) is increasingly used in the treatment of mental diseases, especially acupuncture and Tuina therapy, and has achieved good clinical results.^[12–14]^ At present, there is no systematic review of acupuncture plus Tuina for the treatment of chronic insomnia, so this study will evaluate the efficacy and safety of acupuncture plus Tuina in the treatment of chronic insomnia, and provide evidence for clinical decision-making.

## 2 Methods and analysis

This protocol, which has been reported, is based on the preferred reporting items for systematic reviews and meta-analyses protocols guidelines and the corresponding checklist.^[15]^ This protocol was registered on the international platform of registered systematic review and meta-analysis protocols (INPLASY2021100115).

### 2.1 Inclusion criteria

#### 2.1.1 Types of studies

This study will be included in a randomized controlled trial of acupuncture plus Tuina in the treatment of chronic insomnia. The language of the trial was limited to both Chinese and English. We will exclude nonrandomized controlled trials, review studies, case reports, and animal experiments.

#### 2.1.2 Types of participants

Studies on adult patients diagnosed with chronic insomnia were included in this study. No limitations of location, educational background, and gender were imposed.

#### 2.1.3 Types of interventions

The treatment group was treated with acupuncture plus Tuina, and acupoints and frequency were not limited. At the same time, the control group received oral medication, sham acupuncture, placebo, Chinese herbal medication, physical therapy, and so on, or even without treatment.

#### 2.1.4 Types of outcomes

Sleep efficiency was the primary outcome of chronic insomnia. We also considered the following indexes: PSQI, SDRS, ESS, ISI, SDS, PSG, MSLT, and MWT as secondary outcomes. In addition, we will carefully observe the adverse reactions of patients during acupuncture and Tuinas.

### 2.2 Search methods for the identification of studies

#### 2.2.1 Electronic searches

The following databases will be searched: Web of Science, PubMed, Cochrane Library, Embase, and Medline databases. In addition, we will also collect 4 databases of China: China National Knowledge Infrastructure, China Biomedical Literature Database, VIP Database, and Wan-fang Database. We selected eligible studies published up to October 2021. The search terms were insomnia, anhypnosis, chronic insomnia, vigilance, hyposomnia, acupuncture, Tuina, Tuina therapy, etc. The search strategy for the PUBMED is presented in Table 1. Similar research strategies for other electronic databases have been adapted and applied.

**Table 1:** Search strategy for the PubMed database.

Number	Terms
#1	Insomnia (all field)
#2	Agrypnia (all field)
#3	Sleeplessness (all field)
#4	Aypnia (all field)
#5	Hyposomnia(all field)
#6	Chronic insomnia
#7	#1 or #2–7
#8	Acupuncture(all field)
#9	Acupoint (all field)
#10	Needling(all field)
#11	Acupuncture treatment (all field)
#12	Fire needling (all field)
#13	Scalp acupuncture (all field)
#14	Ear acupuncture (all field)
#15	Intradermal needling (all field)
#16	Auricular acupuncture (all field)
#17	Catgut embedding (all field)
#18	Electroacupuncture (all field)
#19	#8 or #9–18
#20	Tuina (all field)
#21	Knead (all field)
#22	Massotherapy (all field)
#23	Massage (all field)
#24	Massieren (all field)
#25	Massaging (all field)
#26	Manipulation (all field)
#27	#20 OR #21-26
#28	Randomized controlled trial (all field)
#29	Controlled clinical trial (all field)
#30	Random allocation (all field)
#31	Randomized (all field)
#32	Randomly (all field)
#33	Placebo (all field)
#34	Single-blind method (all field)
#35	Double-blind method (all field)
#36	Trials (all field)
#39	#28 or #29-36
#40	#7 and #19 and #27 and #39

#### 2.2.2 Searching for other resources

We will search for a list of related references for additional trials. The PubMed and Cochrane Library will be searched for existing systematic reviews related to our topic to search their reference lists for further studies. We will search a reference list for identifying published journals, books, conference articles, and gray literature related to this research topic. We also retrieved manually related documents, such as replacing and supplementing some reference documents, such as medical textbooks and clinical laboratory manuals, and the World Health Organization International Registry of Clinical Trials.

### 2.3 Data selection process

**Figure 1:**
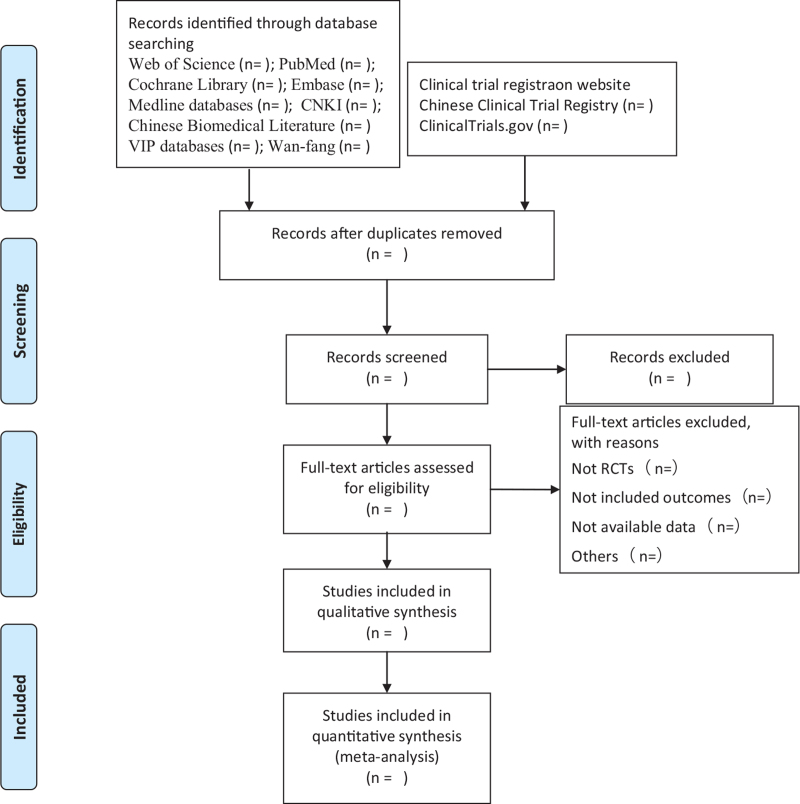
Flow diagram of study selection process.

Researchers will import all the literature into Endnote X9 software for collation, and repetitive studies will be deleted by the software. Two experienced reviewers independently searched all databases, excluded obviously unrelated literature, and deleted duplicated relevant research abstracts. The disagreement will be settled regarding study inclusion by a third reviewer, as necessary. If the information is incomplete, the reviewer should contact the author to obtain complete information and delete the full text of the research review to record the specific reasons for exclusion. Finally, the final included literature was exchanged and checked by researchers. A flowchart of the screening process is presented in Figure 1.

### 2.4 Data extraction and analysis

Our study team built a data extraction table to extract the data of the final included study, including author, year, sample size, course of treatment, intervention measures, outcome indicators, and adverse reactions, among others. If the above data display is incomplete, we will contact the author of the article. If necessary, all reviewers participated in the discussions to resolve the differences.

### 2.5 Assessment of risk of bias in included studies

Two reviewers will evaluate the quality of each article using the Cochrane Collaboration Tool to assess bias risk. The risk table includes 6 items: random sequence generation mode, whether to use allocation concealment, whether to blind the subjects and intervention providers, whether to blind the results evaluators, whether the results data are complete, whether to select the results report, and other bias sources. According to the criteria, it is classified as “low”, “unclear”, and “high”. These even domains will be separately appraised by 2 reviews, and any differences will be resolved through discussion.

### 2.6 Data synthesis and analysis

RevMan 5.4 software will be used for data synthesis and meta-analysis. All studies used the I^2^ value of the chi-square test to determine the heterogeneity. When I^2^ was < 50%, it was considered acceptable. When I^2^ > 50%, a subgroup analysis should be performed to identify the potential causes and record them. When there is homogeneity in the merged outcome results across sufficient studies, a meta-analysis will be conducted. Otherwise, we carried out a subgroup analysis to explore the causes of heterogeneity.

### 2.7 Assessment of reporting biases

In terms of accuracy, we used funnel charts to assess the reporting bias. If there are >10 trials in accordance with the study, we will conduct a test for funnel plot asymmetry using the Egger method.

### 2.8 Analysis of subgroups or subsets

When meta-analysis shows significant heterogeneity, we will perform subgroup analysis according to the type of insomnia and different methods of study quality, intervention, control, and outcome measurements.

### 2.9 Sensitivity analysis

We conducted a sensitivity analysis according to the recommendations of the Cochrane Handbook. Sensitivity analysis was used to test the quality of the research contained in the sampled documents. The main analysis points included the impact of method quality, sample size, and missing data on the study. The stability of the conclusions can be tested by re-analyzing the conclusions by inputting missing data and changing the type of research.

### 2.10 Grading the quality of evidence

It is recommended to use the grading of recommendations, assessment, development, and evaluation to analyze the quality level of evidence.^[16]^ The evaluation will be based on study limitations, inconsistencies, discontinuity, inaccuracy, and publication bias of the study design. Finally, the research quality was divided into 4 levels from high to low: high, medium, low, and very low.

### 2.11 Ethics and dissemination

We used aggregated published data to exclude individual patient data, so ethical approval and informed consent were not required. The results of the system review will be published in peer-reviewed journals or disseminated in peer-reviewed publications.

## 3 Discussion

The main characteristics of insomnia are difficulty starting sleep, difficulty maintaining sleep, or both. Clinical mixed sleep disorders are most common.^[17]^ Since insomnia is a subjective complaint, the cause of insomnia has not reached a consensus, and it is difficult to define and diagnose.^[18]^ The disease for a long time can easily cause physical and mental fatigue, anxiety, depression, and other symptoms. Anxiety, depression, and insomnia can worsen the condition.^[19]^ Western medicine treatment is often benzodiazepine sedative hypnotics, even with psychotropic drugs; however, these drugs have more adverse reactions and are easy to addicted, the application of a long time has poor efficacy, and psychotropic drugs will be a heavier psychological burden for patients.^[20,21]^ In the treatment of insomnia, TCM mainly adjusts Yin and Yang as a whole, which has unique curative effects and few adverse reactions. Acupuncture and Tuina are traditional physical therapies of TCM, which can improve the curative effect, shorten the course of treatment, simplify the operation, have fewer side effects, and have a significant effect on the treatment of chronic insomnia.^[22,23]^ Previous studies have reported that acupuncture plus Tuina can benefit patients with chronic insomnia. However, no systematic review has explored this issue. Therefore, this study is the first to systematically study the efficacy and safety of acupuncture combined with Tuina in the treatment of patients with chronic insomnia. The results of this study provide useful evidence for clinical practice and future research.

## Author contributions

Weiwan Yang and Xiaole Guo contributed to the conception of the study. Weiwan Yang drafted and revised the manuscript. The search strategy was developed by all the authors and performed by Ting Pan and Qi Lu, who also independently screened the potential studies, extracted data from the included studies, assessed the risk of bias, and completed the data synthesis. Haili Wang will arbitrate in cases of disagreement and ensure the absence of errors. All authors approved the publication of the protocol.

ORCID ID https://inplasy.com/inplasy-2021-10-0115/

**Data curation:** Weiwan Yang, Hongfeng Wang.

**Formal analysis:** Xiaole Guo, Qi Lu.

**Funding acquisition:** Hongfeng Wang.

**Investigation:** Qi Lu, Ting Pan.

**Methodology:** Weiwan Yang, Xiaole Guo.

**Validation:** Haili Wang.

**Writing – original draft:** Weiwan Yang.

**Writing – review & editing:** Hongfeng Wang, Xiaole Guo.

## References

[1] Buysse DJ. Insomnia. JAMA 2013;309:706–16. 23423416 10.1001/jama.2013.193 PMC3632369

[2] Cunnington D Junge MF Fernando AT. Insomnia: prevalence, consequences and effective treatment. Med J Aust 2013;199:S36–40. 10.5694/mja13.10718 24138364

[3] Riemann D Nissen C Palagini L Otte A Perlis ML Spiegelhalder K. The neurobiology, investigation, and treatment of chronic insomnia. Lancet Neurol 2015;14:547–58. 25895933 10.1016/S1474-4422(15)00021-6

[4] Javaheri S Redline S. Insomnia and risk of cardiovascular disease. Chest 2017;152:435–44. 28153671 10.1016/j.chest.2017.01.026 PMC5577359

[5] Cipriani G Lucetti C Danti S Nuti A. Sleep disturbances and dementia. Psychogeriatrics 2015;15:65–74. 25515641 10.1111/psyg.12069

[6] Sochal M Małecka-Panas E Gabryelska A. Determinants of sleep quality in inflammatory bowel diseases. J Clin Med 2020;9:2921. 10.3390/jcm9092921 PMC7563861 32927760

[7] Morin CM Drake CL Harvey AG. Insomnia disorder. Nat Rev Dis Primers 2015;1:15026. 27189779 10.1038/nrdp.2015.26

[8] Spielman AJ Caruso LS Glovinsky PB. A behavioral perspective on insomnia treatment. Psychiatr Clin North Am 1987;10:541–53. 3332317

[9] Dopheide JA. Insomnia overview: epidemiology, pathophysiology, diagnosis and monitoring, and nonpharmacologic therapy. Am J Manag Care 2020;26: (Suppl 4): S76–84. 32282177 10.37765/ajmc.2020.42769

[10] Riemann D Spiegelhalder K Feige B. The hyperarousal model of insomnia: a review of the concept and its evidence. Sleep Med Rev 2010;14:19–31. 19481481 10.1016/j.smrv.2009.04.002

[11] Levenson JC Kay DB Buysse DJ. The pathophysiology of insomnia. Chest 2015;147:1179–92. 25846534 10.1378/chest.14-1617 PMC4388122

[12] Lu M Liu X. Insomnia due to deficiency of both the heart and spleen treated by acupuncture-moxibustion and Chinese Tuina. J Tradit Chin Med 2008;28:10–2. 18416075 10.1016/s0254-6272(08)60004-7

[13] Wu XF Zheng XN Wang Y. Acupuncture at acupoints along the meridians for primary insomnia: a multi-center randomized controlled trial. Zhongguo Zhen Jiu 2020;40:465–71. 32394651 10.13703/j.0255-2930.20190430-k0001

[14] Zhang L Tang Y Hui R. The effects of active acupuncture and placebo acupuncture on insomnia patients: a randomized controlled trial. Psychol Health Med 2020;25:1201–15. 32167794 10.1080/13548506.2020.1738015

[15] Moher D Shamseer L Clarke M. Preferred reporting items for systematic review and meta-analysis protocols (PRISMA-P) 2015 statement. Syst Rev 2015;4:01. 10.1186/2046-4053-4-1 PMC4320440 25554246

[16] Langer G Meerpohl JJ Perleth M. GRADE guidelines: 12. Developing summary of findings tables—dichotomous outcomes. Z Evid Fortbild Qual Gesundhwes 2013;107:646–64. 24315336 10.1016/j.zefq.2013.10.034

[17] American Academy of Sleep Medicine. International classification of sleep disorders. 3rd ed. Darien: American Academy of Sleep Medicine; 2014.

[18] Jaussent I Morin C Dauvilliers Y. Definitions and epidemiology of insomnia. Rev Prat 2017;67:847–51. 30512811

[19] Blake MJ Trinder JA Allen NB. Mechanisms underlying the association between insomnia, anxiety, and depression in adolescence: Implications for behavioral sleep interventions. Clin Psychol Rev 2018;63:25–40. 29879564 10.1016/j.cpr.2018.05.006

[20] Liu MT. Current and emerging therapies for insomnia. Am J Manag Care 2020;26: (Suppl 4): S85–90. 32282178 10.37765/ajmc.2020.43007

[21] Welsh JW Tretyak V McHugh RK Weiss RD Bogunovic O. Review: adjunctive pharmacologic approaches for benzodiazepine tapers. Drug Alcohol Depend 2018;189:96–107. 29906718 10.1016/j.drugalcdep.2018.04.028

[22] Poon MM Chung KF Yeung WF Yau VH Zhang SP. Classification of insomnia using the traditional Chinese medicine system: a systematic review. Evid Based Complement Alternat Med 2012;2012:735078. 22899958 10.1155/2012/735078 PMC3414091

[23] Hong J Chen J Kan J Liu M Yang D. Effects of acupuncture treatment in reducing sleep disorder and gut microbiota alterations in PCPA-induced insomnia mice. Evid Based Complement Alternat Med 2020;2020:3626120. 33178314 10.1155/2020/3626120 PMC7647758

